# Roadside increments in PM_10_, NO_x_ and NO_2_ concentrations observed over 2 months at a major highway in New Zealand

**DOI:** 10.1007/s11869-014-0305-4

**Published:** 2014-11-16

**Authors:** Ian Longley, Elizabeth Somervell, Sally Gray

**Affiliations:** National Institute of Water and Atmospheric Research (NIWA) Ltd, Private Bag 99940, Auckland, 1149 New Zealand

**Keywords:** Traffic-related pollution, Roadside increment, Nitrogen dioxide, Particulate matter, Oxides of nitrogen

## Abstract

Continuous and simultaneous observational particulate matter (measured as PM_10_), nitrogen dioxide (NO_2_) and oxides of nitrogen (NO_x_) data were captured at a kerbside site alongside a major highway in Auckland, New Zealand, and at a pair of setback sites within 250 m of the highway, day and night over 8 weeks. The three measurement sites were intended to allow emissions from the highway to be largely isolated from other sources. By filtering the data and subtracting upwind concentrations, the average roadside increment was calculated to be 1.8, 7.2 and 101.4 μg m^−3^ for PM_10_, NO_2_ and NO_x_, respectively, relative to a predominantly upwind setback site, and −0.1, 9.4 and 98.5 μg m^−3^ for PM_10_, NO_2_ and NO_x_, respectively, relative to a downwind setback site. The negative value for PM_10_ was attributed to local evening heating sources impacting the setback site. On days when peak 24 h PM_10_ concentrations were observed, the absolute kerbside increment was 2.1 μg m^−3^. The absolute roadside 24 h average PM_10_ increment varied diurnally, peaking (on average) at 2.4 μg m^−3^ during peak traffic hours. The largest observed 24-h average PM_10_ roadside increment was 6.9 μg m^−3^ and exceeded 5 μg m^−3^ on nine occasions. On each of these occasions, the daily mean wind speed was less than 2 m s^−1^. The diurnally averaged difference in NO_x_ concentrations between the kerbside site and the setback sites clearly resembled the diurnal cycle in traffic volume, and peaked during the morning traffic peak at around 180 μg m^−3^. Background NO_x_ concentrations were slightly higher in our study compared to a similar study in Las Vegas but absolute roadside concentrations were higher. This may be consistent with higher NO_x_ emission factors in Auckland, but differences in the precise distance of the monitor from the road lanes and differences in meteorology need to be considered.

## Introduction

Long-term spatial gradients in air pollutants concentrations exist within cities at the ~100-m scale, and those intra-city gradients are often as significant, if not more so, than inter-city gradients. An important example is traffic-related air pollutants whose concentrations can be elevated within 150 m of a major road by up to 5 times (Karner et al. 2010).

The distance between residence and major roads has a significant association with a wide range of adverse health outcomes (HEI 2010). Including these localised gradients increases the estimated burden compared to conventional airshed-scale analyses (e.g. Jerrett et al. 2005). The mechanisms underlying these risks are still poorly understood but regulatory agencies are charged with managing them on a precautionary basis.

In lieu of health-based guidance specifically relating to roadside locations, regulatory agencies often use standards and guidelines to represent health-endangering concentrations of air pollution. The WHO Guideline of 40 μg m^−3^ of nitrogen dioxide (NO_2_) as an annual mean is widely used to judge the health significance of traffic-related air pollution because it is accepted that road traffic emissions are the major source of NO_2_ concentrations in many urban areas.

At roadside locations, the contribution of the adjacent road as distinct from other sources is not easily determined. For example, Engler et al. (2012) attributed 58–73 % of particulate matter (PM_10_) concentrations measured at a roadside site in Leipzig to ‘regional’ sources, but also concluded that urban background concentrations were equal to rural concentrations. Thus, how much of the remaining 27–42 % was due to emissions from the road immediately adjacent to the roadside site, from all of the roads in Leipzig or from other sources was unclear. Accurately identifying the sources of the roadside increment is vital for the purposes of air quality management as each source requires different mitigation strategies.

Looking to the future, urban planning tools for air quality and public health risk management are increasingly being explored. Recently, Perez et al. (2012) showed how reducing the population of children exposed to the roadside concentration increment could be a substantial and viable public health intervention, even if combined with increasing urban intensification (and especially if combined with clean vehicle strategies and land-forms which reduce vehicle-kilometres-travelled).

All of these issues—epidemiological research, health risk assessment, risk management and urban planning guidance—can be better informed by empirical data that quantifies the ‘roadside increment’, i.e. the increase in concentrations at the roadside relative to the urban background, and the ‘corridor width’, i.e. the lateral distance over which a roadside increment is observable. Previous attempts to quantify the corridor width have generally consisted of measured gradients at increasing lateral distances from a road. The meta-analysis by Karner et al. (2010) revealed some general patterns, but substantial variation, partly due to the use of arbitrary ‘kerbside’, or ‘setback’ reference points with an often unknown relationship to the local urban background. Many studies have relied on existing or long-term monitoring where measurements are only made on one side of a road, making it difficult to distinguish the subject road’s emissions from all other (background) sources (e.g. Engler et al. 2012). A small number of campaign-based studies have included monitoring simultaneously on both sides of the subject road (e.g. Zhu et al. 2002, 2004, 2006, 2009; Clements et al. 2009; Pirjola et al. 2006; Beckerman et al. 2008; Baldauf et al. 2008; Hagler et al. 2009). However, a common weakness of many campaign-based studies is their relatively short duration relative to the temporal variation in concentrations often observed at long-term monitoring sites.

Recently, a major study has filled this gap. Continuous measurements were made at four sites along a transect crossing the I-15 freeway in Las Vegas: one site 100 m to the east (upwind) and three, at 20, 100 and 300 m from the roadside to the west (downwind) for a whole year (Kimbrough et al. 2012). The mean roadside increment was 5 ppb for NO_2_, 16 ppb for oxides of nitrogen (NO_x_), 0.07 ppm for carbon monoxide (CO) and 0.74 μg m^−3^ for black carbon (BC)—or 29, 53, 26 and 95 %, respectively. Kimbrough et al. (2013) expanded this analysis by filtering the data for westerly winds only and Henry et al. (2011) presented a non-parametric trajectory analysis, to further refine estimates of roadside increment. However, it is unclear to what degree such increments and gradients may be applicable in different climates or in locations with different traffic volumes or average vehicle fleet emissions. Furthermore, Baldauf et al. (2013) indicated how the location of the I-15 freeway in a cutting may have reduced roadside concentrations reported by Kimbrough et al. (2012, 2013) relative to an at-grade road segment.

Auckland, New Zealand is one location with a different climate to Las Vegas, having relatively high winds and a low prevalence of calms. It also has a substantially different vehicle fleet with many imported used vehicles from the Far East with an average age over 10 years old and a large number of older trucks with unknown emissions. This paper describes results from an observational campaign conducted in Auckland. The analysis presented here focusses on variability in the roadside increment, particularly as a function of wind direction. Results from other aspects of the study, including roadside ozone data, passive and mobile monitoring in the wider surrounding neighbourhoods, modelling of background air quality and implications for exposure assessment for local residents are presented elsewhere (Elangasinghe et al. 2014; Pattinson et al. 2014a, b, c, Pattinson et al. *submitted*, Longley et al., *in preparation*).

## Materials and methods

### Study area

#### Study area location and characteristics

**Fig. 1:**
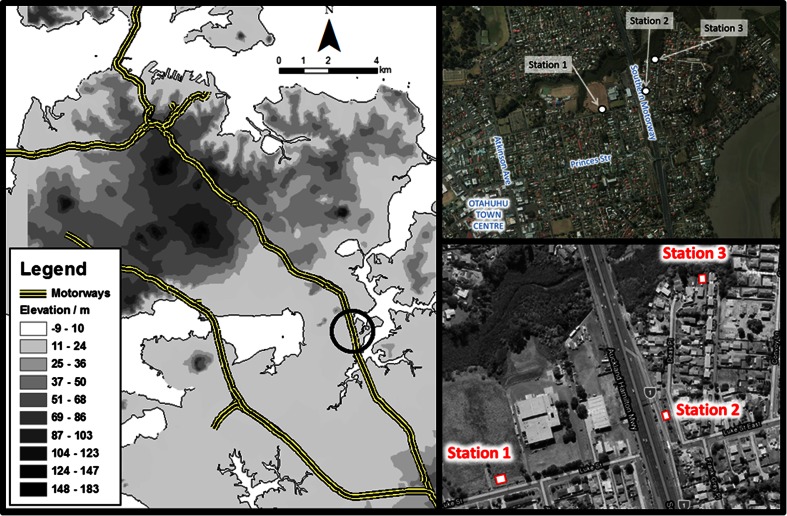
*Left* location of the study area (Otahuhu East, *circled*) in Auckland, indicating local physical topography. *Right* satellite image of the study area indicating the three continuous air quality monitoring stations. The study area predominantly contains detached low-rise residential dwellings with commercial land-use in Otahuhu town centre

The study area selected was in Otahuhu East in Auckland, New Zealand (Fig. 1). Otahuhu East is located on a narrow (approximately 3 km wide) isthmus connecting central Auckland with south Auckland. The study area is relatively flat with an altitude of <20 m, centred on a central reference point of at 36.93611° S, 174.85252° E.

The Otahuhu East study area is bisected by the Auckland Southern Motorway (state highway 1 or SH1) which travels approximately north to south (see Fig. 1). The local area is linked to the motorway by a single interchange with Princes Street. Land-use is predominantly residential, consisting mostly of single storey detached dwellings, most of which possess private gardens.

Data collected at Auckland Airport, 10 km south-west of the study area, shows that the predominant wind direction is south-westerly, with a secondary mode of north-easterly winds. However, in the case of calmer winds, which are more significant for air quality, the prevalence of south-westerly and north-easterly winds is more similar.

Traffic data for this section of the motorway was provided by the New Zealand Transport Agency (http://www.nzta.govt.nz/resources/state-highway-traffic-volumes/), from permanently installed counters covering all lanes of the motorway, including ramps at the Princes Street interchange. During the year of our study (2010), annual average daily traffic (AADT) on the motorway through Otahuhu was 116,000 north of the Princes Street interchange, and 122,000 south of the interchange. Using similar NZTA data, we estimate that only 24 km of motorway in Auckland has volumes of this magnitude or greater. Traffic data for local roads in the study area is provided by Auckland Transport and is based on 1-week manual counts conducted once every 3 years. The AADT on Princes Street is approximately 13,000. On all other roads in the study area, the AADT is <2000.

Other than road traffic sources, the only other known emission sources in the study areas include domestic heating and cooking. Non-electric domestic heating sources in Auckland are from wood-burning and gas appliances. Domestic home heating sources can impact PM_10_ levels across Auckland from May to August between 6 pm and 6 am, with levels peaking around 11 pm to midnight; however, this signal is often indistinct except on colder nights with low wind speeds. The impact of cooking sources on ambient concentrations of air pollutants has not been documented in Auckland.

**Fig. 2:**
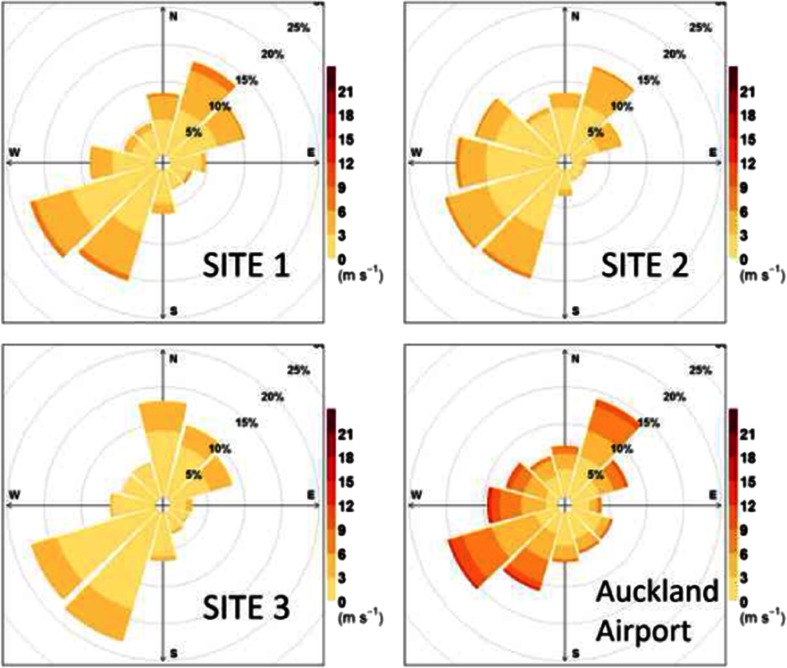
Windroses for the campaign meteorological sites, plus Auckland Airport, during the period of the continuous observational campaign

There are two significant industrial point sources within 3 km of the study area. There is a gas turbine electricity generating station 1.7 km to the southeast which, according to the Auckland Council emission inventory, is consented to emit 606 t/year of NO_x_ and 1.38 t/year of PM_10_. However, the very low prevalence of south-easterly winds (Fig. 2) means that an influence of the generating station on the study area is not expected. The other major source is a small steelworks 2.9 km to the west-south-west of the study area, consented to emit 21.2 t/year of PM_10_ and 69.9 t/year of NO_x_. Neither of these point sources have previously been identified as causing local air quality problems.

### Instruments and methods

Although road traffic is known to be responsible for the emissions of many different air pollution species, oxides of nitrogen (NO_x_) were chosen to be the primary indicator and main focus of our study. This is because the technology for monitoring NO_x_ is well-developed and widely used, road traffic is the dominant source of NO_x_ emissions in most urban areas and NO_x_ instruments have a high sensitivity relative to the concentrations and concentration gradients observed in roadside locations. We also included assessments of PM_10_ and nitrogen dioxide (NO_2_) due to the existence in New Zealand of National Environmental Standards for both, and the widespread use of both indicators in health risk analysis.

The core of our design was the establishment of three fixed continuous monitoring sites, one at the kerbside and two at setback sites, one on either side of the road. The three sites were to be established, as far as practicalities allowed, to roughly represent three points along a trajectory aligned with the predominant wind direction, such that one site was upwind and two were downwind. In this design, we make the assumption that there is no source or sink between the upwind and downwind sites other than the road source. The result of this design is that the contribution of the road to local air quality can be estimated from the difference in concentrations between downwind and upwind sites.

Fixed monitoring sites were installed at three locations, one on the west of SH1 and two on the east side, as illustrated in Fig. 1, and listed here from west to east:

- Station 1, on undeveloped land on Luke Street, ~250 m west of SH1
- Station 2, on Deas Place Reserve, immediately adjacent to the southbound Princes Street off-ramp of SH1
- Station 3, in the rear yard of a private property on Deas Place ~150 m east of SH1

At each of these stations, there was a custom-built air conditioned trailer, with instruments to measure NO_x_, NO_2_, and PM_10_. At Stations 1 and 2, a 3-m inlet was used to sample PM_10_ from a height of 4.2 m (1.4 m above the trailer roof), whilst NO_x_ was sampled at a height of 3.75 m (0.95 m above the trailer roof). At Station 3, PM_10_ was sampled through a 1-m inlet at a height of 3.85 m whilst NO_x_ was sampled at a height of 3.4 m (0.5 m above the trailer roof).

Each site had its own independent meteorological observations, with masts at heights of 9.5 m at Station 1, 6.0 m at Station 2 and 7.5 m at Station 3. In our analysis, the wind data have not been adjusted for these differences in height.

Instrumentation is listed in Table 1. Where possible, monitoring was conducted to comply with the applicable Australian/New Zealand Standard. Some requirements of these standards could not be met due to the short time frame of this study. The NO_x_ analyser was operated at a flow of 0.5 l/min and a range of 0–1000 ppb. Five-point instrument calibrations for NO and NO_2_, including zero and span cheques, were performed on-site at the start and end of the campaign and on a monthly schedule using certified gas cylinders and an API M700 calibrator. No weekly performance cheques were undertaken but the instrument display was checked on each visit to the site.

**Table 1:** Instruments designated to fixed-point monitors

Contaminant	Instrument	Precision
PM_10_	Thermo FH62C14 Beta Attenuation Monitor	±2 μg m^−3^ (over 24 h)
NO_2_, NO	API Model 200 chemiluminescence analyser	±0.5 %
Wind speed	Vector A101M	±0.1 m s^−1^
Wind direction	Vector W200P wind vane	±2°
T and RH	Vaisala 50Y	±5 %
Solar radiation	Licor L200	
Rainfall	Ota tipping bucket 0.2 mm tip	

The beta attenuation monitor (BAM) was operated at 16.7 l/min on a range −100 to 900 μg m^−3^. Flow cheques were undertaken on-site at least every month and were found to be consistent throughout the campaign with no adjustment necessary.

The complete observational campaign began on 2 April 2010 and finished on 29 September 2010. The commissioning and decommissioning of the three fixed sites was staggered for logistical reasons. Results reported in this paper relate only to the periods when all three sites provided quality assured data. For NO_2_, NO_x_ and meteorological data, this was from 14 May–10 June and from 30 June–3 August 2010. For PM_10_, this was from 27 May–20 June and from 30 June–4 August 2010. Within these dates, monitoring was continuous for 24 h a day, 7 days a week.

An instrument co-location exercise prepared at the end of the campaign at Station 2 had to be abandoned when the trailer’s air conditioning failed. Two of the BAMs used in this study (at Stations 1 and 3) were subsequently co-located in a different campaign over a period of 1 month, using the same inlet arrangement as in this study. A good linear fit (*R* ^2^ = 0.96) with a slope of 0.99 was found. Consequently, none of the BAM data in this study was adjusted.

The raw data from the NO_x_ and the beta attenuation monitors, as well as from the meteorological sensors, were sampled every 3 s and logged as a 10-min average on Campbell CR10X data loggers. The data were downloaded from the loggers via cell phone telemetry and checked on each working day. Any invalid data were removed and a comment was included in the metadata file to explain why they were taken out. For the gaseous data, the results from the calibrations were used to correct the 10 min data and the resulting value was used to determine the hourly and 24 h average data. For the PM_10_ data, the 10 min data was averaged to hourly and 24 h data.

Measurements of CO were also made at each of the three stations during the campaign. However, the CO monitor at kerbside Station 2 reported a fault very early in the campaign. The fault could not be diagnosed at the time but was identified during the quality assurance process. It was determined that the data recorded did not meet the quality standards required, nor could it be adequately corrected. This prevents us from considering roadside increments of CO and consequently, do not report any CO data in this paper. Measurements of O_3_ were also made at Station 1 and 2, but are not presented in this paper.

### Contribution of the motorway to roadside concentrations—method

Key to our study design was the measurement of the same parameters using the same instruments simultaneously both upwind and downwind of the motorway. To estimate the contribution of the motorway to roadside concentrations, we made the assumption that the only significant emission source between the three monitoring sites is the traffic on the motorway. The motorway contribution is then equal to the difference between upwind and downwind concentrations. The validity of this assumption will be investigated within the course of the following analysis.

In order to qualify the up/downwind location of a site, data have been segregated into westerly and easterly winds. We defined the ‘westerly’ subset as data for which all three meteorological sites reported wind directions in the range 180 to 330° and wind speed was >1 m s^−1^. Similarly, we defined the ‘easterly’ subset as data for which all three sites reported wind directions in the range 0–150° and wind speed was >1 m s^−1^.

In order to consider the potential contribution of motorway emissions to exceedance of the National Environmental Standards (NO_2_ 200 μg m^−3^ as a 1-h average, PM_10_ 50 μg m^−3^ as a 24-h average), we consider the hourly (NO_2_) and 24-h (PM_10_) difference in concentrations between Station 2 and Station 1 or 3.

## Results

### Observed meteorological conditions during the campaign

Station 1 possessed the taller meteorological mast and was the furthest station from buildings and trees. Consequently, we take Station 1 to provide the most generally representative meteorological data of the three stations. The range of meteorological conditions observed at Station 1 are summarised in Table 2. Figure 2 shows the campaign wind roses from Stations 1–3 and Auckland Airport (10 km to the south-west), indicating winds largely conformed to the expected climate norm of predominant south-westerly winds, but with significant prevalence of north-easterly winds and lesser prevalence of other wind directions. Rainfall was observed during 335 h (17 % of the campaign).

**Table 2:** Meteorological summary statistics of hourly average data during the campaign (based on Station 1)

Parameter	Min	Median	Mean	Max	Interquartile range
Temperature (°C)	1.9	12.4	11.9	19.1	10.0–14.2
Relative humidity (%)	36	81	76	88	67–82
Wind speed (m s^−1^)	0.0	2.1	2.4	7.6	1.1–3.5
Solar radiation (W m^−2^)	0.0	0.0	88	608	0–132
Rainfall (mm)	0.0	0.0	0.2	21.8	0–0

### Average contribution of the motorway to roadside concentrations

Summary statistics of hourly air quality concentrations are provided in Table 3. The average absolute concentrations at the motorway’s edge (Station 2) were 190.9 μg m^−3^ for NO_x_, 26.1 μg m^−3^ for NO_2_ and 18.7 μg m^−3^ for PM_10_.

**Table 3:** Statistical summaries of hourly concentrations (μg m^−3^)

	NO_x_				NO_2_				PM_10_			
Parameter	Mean	Median	Max	Interquartile range	Mean	Median	Max	Interquartile range	Mean	Median	Max	Interquartile range
Station 1	90.5	39.3	864.9	19.0–110.2	18.9	17.8	46.4	10.4–26.2	17.0	14.1	93.6	9.3–20.7
Station 2	190.9	151.2	1171.0	32.9–278.1	26.1	27.3	80.8	12.4–37.8	18.7	16.1	93.5	9.8–23.5
Station 3	92.4	43.0	850.0	12.0–124.4	16.6	16.9	54.9	7.2–24.1	18.8	15.4	95.1	9.4–24.6

Mean NO_x_ concentrations at the roadside Station 2 were approximately 100 μg m^−3^ higher than at both Station 1 and Station 3. Mean NO_2_ concentrations at Station 2 were 7.2 μg m^−3^ higher than at the 250-m (west) setback site of Station 1 and 9.4 μg m^−3^ higher than at the 150-m (east) setback site at Station 3. Mean PM_10_ concentrations at Station 2 were 1.8 μg m^−3^ higher than at the western setback site, but 0.1 μg m^−3^ lower than at the eastern setback site.

**Fig. 3:**
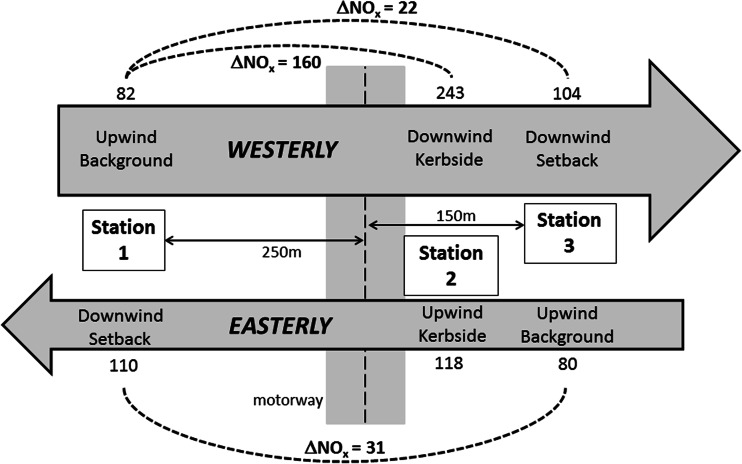
Visualisation of campaign mean NO_x_ concentrations (μg m^−3^) in westerly winds (*above*) and easterly winds (*below*)

**Fig. 4:**
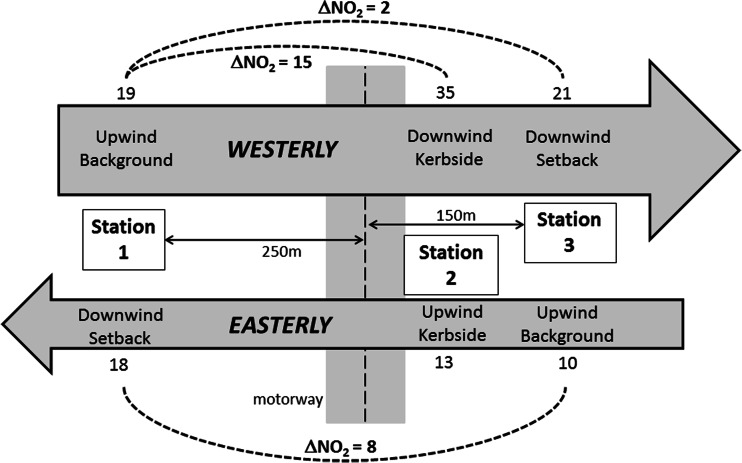
Visualisation of campaign mean NO_2_ concentrations (μg m^−3^) in westerly winds (*above*) and easterly winds (*below*)

**Fig. 5:**
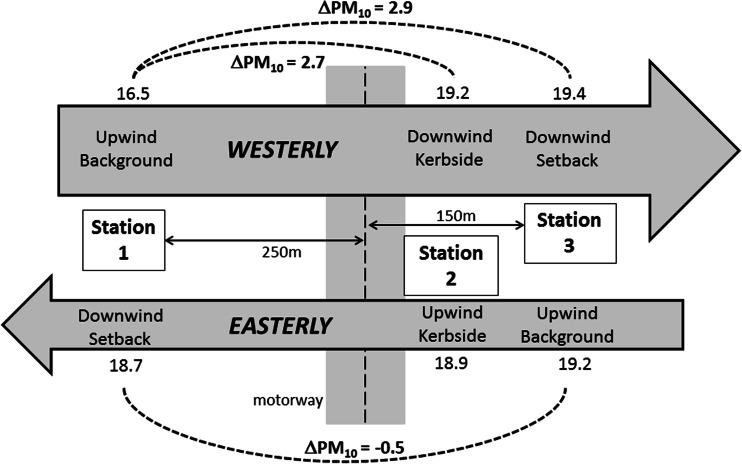
Visualisation of campaign mean PM_10_ concentrations (μg m^−3^) in westerly winds (*above*) and easterly winds (*below*)

Figures 3, 4 and 5 provide a visualisation of this data. In these figures, data are separated in easterly and westerly wind directions. The numbers represent mean concentrations at each site for each wind sector. Also shown are the mean differences between kerbside and setback stations for each wind sector.

The mean differences between sites as a function of wind sector were also presented in Table 4. The upwind concentrations in westerly and easterly winds are similar for NO_x_, higher in westerlies for NO_2_ and higher in easterlies for PM_10_. In easterly winds, concentrations of NO_x_, and NO_2_ were elevated at the kerbside site relative to the setback sites despite it being on the upwind side of the motorway.

**Table 4:** Mean hourly concentrations (μg m^−3^) for each station, and differences between stations, including data filtered by hourly vector-averaged wind direction

	NO_x_			NO_2_			PM_10_		
Parameter	All data	Westerly	Easterly	All data	Westerly	Easterly	All data	Westerly	Easterly
Station 1	90.5	82.3	110.8	18.9	19.5	18.3	17.0	16.5	18.7
Station 2	190.9	242.6	118.3	26.1	34.5	13.4	18.7	19.2	18.9
Station 3	92.4	104.2	79.5	16.6	21.2	9.8	18.8	19.4	19.2
Station 2–Station 1 (kerbside–west setback)	101.4	160.3	7.5	7.2	15.1	−4.9	1.8	2.7	0.2
Station 2–Station 3 (kerbside–east setback)	98.5	138.3	38.8	9.4	13.3	3.6	−0.1	−0.2	−0.4
Station 3–Station 1 (east setback–west setback)	1.9	21.9	−31.3	−2.3	1.7	−8.5	1.8	2.9	0.5

**Fig. 6:**
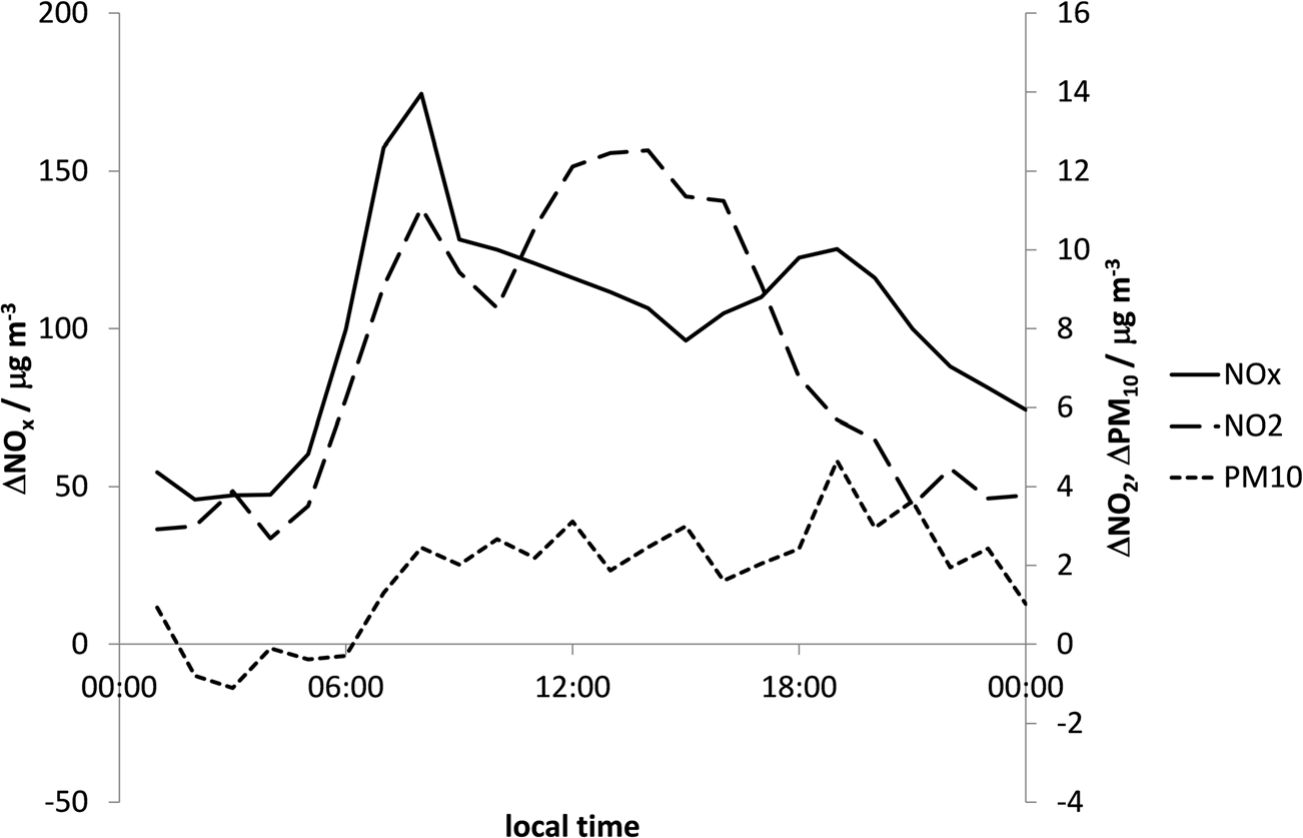
Diurnal average difference in concentrations between Station 2 and Station 1 (i.e. the roadside increment) over the whole campaign (all wind directions)

Hour-by-hour diurnal average NO_x_ concentrations at Station 1 and Station 3 were very similar, whereas NO_2_ concentrations at Station 1 were 1–5 μg m^−3^ higher than at Station 3 on average. Figure 6 shows the diurnally averaged difference in concentrations between the kerbside site of Station 2 and setback site of Station 1 for all wind directions. The diurnal variation in the NO_x_ roadside increment closely resembles the diurnal cycle in traffic volume, and peaks during the morning traffic peak at around 175 μg m^−3^. The NO_2_ roadside increment partially resembled the diurnal cycle in traffic volume, but peaked during the early afternoon at 12 μg m^−3^. The diurnally averaged difference in PM_10_ concentrations between the kerbside site of Station 2 and Station 1 averaged 2.4 μg m^−3^ during the daytime (7 am to 6 pm). The cycle partially resembles the diurnal cycle in traffic volume, but with the difference in PM_10_ between the two sites persisting beyond the evening traffic peak and remaining above zero until 2 am.

### Peak contribution of the motorway to roadside concentrations

The largest value of the difference between hourly average NO_2_ at Station 2 and either setback station was 74.8 μg m^−3^; however, this appears to be an outlier as it relates to a data point recorded at 3 am when the Station 1 concentration was 4 μg m^−3^. The second largest was 46 μg m^−3^, and all but four hourly values were below 40 μg m^−3^. The size of the hourly NO_2_ roadside increment (Station 2–Station 1) was relatively independent of background concentration (Station 1 or 3) such that a roadside increment of 0–40 μg m^−3^ could be observed at almost any time during daylight hours.

Table 5 shows the mean, maximum and 99.9th percentile of 24 h average PM_10_ concentrations and differences between sites. It should be noted that the mean increment between the kerbside (Station 2) and eastern setback site (Station 3) is negative. In fact, on 80 % of the days, this setback site had higher 24-h average PM_10_ concentrations than the kerbside site, indicating the influence of a larger, non-motorway source. By contrast, the western setback site (Station 1) experienced PM_10_ concentrations higher than the kerbside for 26 % of the time.

**Table 5:** Observed 24 h average PM_10_ (μg m^−3^) during the campaign

Site	24 h average PM_10_		
	Mean	Max	99.9th percentile
Station 1	16.8	43.5	43.1
Station 2	18.5	43.4	43.1
Station 3	20.5	45.4	45.2
Station 2–Station 3	−1.9	4.1	4.0
Station 2–Station 1	1.7	6.8	6.8
Station 3–Station 1	3.6	8.3	8.4

The largest observed difference in 24 h PM_10_ concentrations between the kerbside and either setback site was 6.8 μg m^−3^. The difference exceeded 5 μg m^−3^ relative to Station 1 on nine occasions. On each of these occasions, the daily mean wind speed observed on-site was less than 2 m s^−1^.

## Discussion

### The roadside corridor

Many previous roadside observational studies have been conducted over a week, or less. The data used here consisted of continuous observations, day and night, over 8 weeks. The full campaign (for which partial data are available but not presented here) lasted 25 weeks. Although neither as long nor complete as the Las Vegas I-15 study (Kimbrough et al. 2012), our study is nevertheless one of the larger currently reported.

Most roadside air quality studies are based upon the concept of the roadside corridor—a strip of land around a major road in which concentrations of traffic-related air pollutants are elevated above background levels. Some studies have sought to explicitly evaluate the width of that corridor, whilst corridor width can also be inferred from other studies. Two recent reviews have sought to do this (Zhou and Levy 2007; Karner et al. 2010) pooling data from 33 to 41 studies, respectively. Both reviews have highlighted the technical difficulties inherent in doing this due to (a) differences in study design and the way results are reported, (b) difficulties in establishing what the background is and (c) sensitivity to the definition of the corridor edge, considering that that edge is gradual and continuous rather than distinct. Nevertheless, a value of around 150 m for passive pollutants is consistently reported from studies in many different locations.

A key question in interpreting the data in our project is to establish whether the setback fixed monitoring sites, at 150 m east and 250 m west of the motorway, lie within or outside the motorway’s corridor of influence. We cannot rely on other continuous monitors at a greater distance to provide a comparison, as the next nearest monitors are >5 km to the east. On average, concentrations of NO_x_ at both setback sites were approximately equal and substantially lower than at the kerbside site, despite the setback sites being different distances from the motorway. However, if the data is screened by wind direction average NO_x_ concentrations at the downwind site were 21.9 and 31.3 μg m^−3^ higher than the upwind site in westerly and easterly winds, respectively (see Table 3). In the case of westerly winds, for which we have downwind kerbside data, concentrations at the downwind setback site were 12 % of those at the kerbside, once upwind concentrations are subtracted. This suggests that, consistent with international evidence, our fixed sites were at the outer edge of the roadside corridor, and thus represented ‘setback’ rather than ‘roadside’ locations in terms of passive pollutants (NO_x_, particulate mass concentrations).

A distinctive feature of the PM_10_ data was elevated concentrations at Station 3 relative to expectations based on the study design assumption of the sites being primarily influenced by emissions from the motorway. PM_10_ concentrations at all sites were elevated (on average) during the evening hours (1800 hours to midnight). On average, PM_10_ concentrations at the eastern setback site were 1.8 μg m^−3^ higher than at the western site, but this difference ranged from 2 to 4 μg m^−3^ between 1800 hours and midnight, −4 to 0 μg m^−3^ during the morning traffic peak and 0–2 μg m^−3^ at other times. This difference was also sensitive to wind speed, peaking at low winds. We find that our results are consistent with a local domestic nocturnal heating source influencing results at all three stations, but more strongly at Station 3 than elsewhere. This is consistent with Auckland Council’s domestic heating emissions inventory which suggests a higher density of wood-burning emissions in the vicinity of Station 3 than the other Stations. This illustrates how an accurate determination of the roadside increment for PM_10_ can be complicated by the difficulty in establishing a representative reference.

### Absolute contribution of motorway emissions to local air quality

On average, we estimated that the motorway contributed an additional 98.5–101.4 μg m^−3^ to NO_x_, 7.2–9.4 μg m^−3^ to NO_2_ and −0.1–1.8 μg m^−3^ to PM_10_ at the kerbside site above the setback sites. In terms of the potential contribution to exceedances of short-term air quality standards, the peak contribution from the motorway was up to 46 μg m^−3^ in terms of 1-h average NO_2_ concentrations (when one outlier is removed) and up to 6.8 μg m^−3^ in terms of 24-h average PM_10_ concentrations. Our kerbside site was ~5 m from the nearest lane of traffic and ~20 m from the nearest main carriageway lane.

Traffic volumes on the I-15 in the Las Vegas study of Kimbrough et al. (2012) were reported as 205,000 per day compared to 120,000 at our Auckland site. On that basis alone, one might expect a proportionally higher roadside increment in Las Vegas. Kimbrough et al. (2012) indicated that roadside NO_x_ concentrations 20 m east (predominantly downwind) of the freeway were 16 ppb (~34 μg m^−3^) higher than at a point 100 m west (upwind) of the freeway. The equivalent difference in our study was three times higher at ~100 μg m^−3^. When restricted to the predominant wind direction (westerly in both studies), the difference was 31 ppb (63 μg m^−3^) in Las Vegas and 138.3 μg m^−3^ (2.2 times higher) in Auckland.

Further work is required to compare the vehicle emission rates between Auckland and Las Vegas. However, we note that a large proportion of this difference in increment could be attributed to the precise location of the roadside stations in each study. We estimate that both the I-15 and SH1 are ~30 m wide from centreline to edge at the respective monitoring sites. However, whereas the I-15 station was a further 20 m from the road edge (~30 m from the centre of the nearest traffic lane), our site was no more than 5 m from the edge (~20 m from the centre of the nearest main carriageway lane and ~8 m from the centre of the off-ramp lane). This close to the emissions source, concentration gradients can be substantial, making direct comparisons between studies difficult.

Also, we must acknowledge that dispersion conditions are likely to have been different between Auckland in autumn/winter and Las Vegas. Henry et al. (2011) note that ‘Low wind speeds were quite common in these data: the distribution of wind speeds at station 2 was highly skewed with a peak (mode) at 1.3 m s^−1^. The wind rose reported by Kimbrough et al. (2012) notes 7.04 % calms, compared to 0.1 % in our study (Fig. 2). Combining the higher traffic volumes and potentially less efficient dispersion in Las Vegas implies that we would expect a higher contribution to NO_x_ concentrations from the I-15 than SH1. The fact that we appear to observe the opposite may imply higher NO_x_ emission factors in Auckland compared to Las Vegas. The detailed data captured in both studies (and probably others) should facilitate a comparative study around these issues.

In easterly winds, during which our kerbside site was on the predominantly upwind side of the motorway, we nevertheless found elevated concentrations for gases at the kerbside site relative to the upwind setback site, specifically an average elevation of 38.8 μg m^−3^ for NO_x_ (48 %) and 3.6 μg m^−3^ for NO_2_ (36 %). To qualify for inclusion in this calculation, an hourly concentration average had to occur in an hour during which the vector average wind direction at all three stations was within the range 0–150°. Clearly within an hour characterised as ‘easterly’ overall, transient periods of westerly winds could occur, and there were periods when the kerbside Station 2 reported westerly winds when the other two stations reported easterly winds (Fig. 2). Baldauf et al. (2008) have previously reported elevated kerbside concentrations when the site is apparently upwind of the road. Our study was insufficiently instrumented to confirm if traffic-induced turbulence was a significant process in elevating kerbside concentration or if the phenomenon is solely due to inaccurate description of wind direction at the measurement point, especially in conditions likely to lead to meandering flows.

Davy et al. (2010) used a receptor modelling chemical source apportionment technique based on 3 years of filter data to estimate that motor vehicles contribute 2.5–7.0 μg m^−3^ of PM_10_ at five sites across Auckland with varying degrees of local traffic influence. These estimates are not directly comparable to ours as they refer to the contribution from all roads in the region, rather than merely the adjacent road. None of the five sites considered by Davy et al. (2010) are particularly similar to our site but these results do indicate that the adjacent road probably contributes half or less of the total motor vehicle emission load at a typical roadside site.

Comparisons for PM are available from more studies, but a strong caveat needs to be made that these studies involve not only further variability in local meteorology, traffic fleets and monitor siting details, but also variability in measurement technology. We estimated an average contribution of the Auckland Southern Motorway to PM_10_ of 1.8 μg m^−3^, determined from the difference in concentrations between the kerbside and western setback site. An increment of 2.1 μg m^−3^ over a comparable distance was reported for PM_2.5_ by Zhu et al. (2002) downwind of the I-405 freeway in Los Angeles. The I-405 is one of the busiest roads in the world with annual average daily volumes in excess of 300,000. Similarly, Reponen et al. (2003) reported an increment of 2.0 μg m^−3^ in PM_2.5_ between 80 and 400 m downwind of the I-71 freeway in Cincinnati.

We found that the maximum contribution of the motorway as a 24-h midnight-to-midnight average was 6–7 μg m^−3^. We also found a tendency for peak values to occur on days with extended periods of low winds, but not all periods of low winds led to increased motorway contribution. This implies that whether low winds led to increased motorway contribution was likely to be a matter of timing—i.e. whether the low winds coincided with periods of high or low emissions and whether such periods were confined to a single day, or split over 2 days, thus their contribution to 24 h PM_10_ being split between 2 days. Further analysis of the dataset could provide some insight into the climatology of PM_10_ peaks.

### Relative contribution of motorway emissions to local air quality

On average, we estimated that the ratio of mean kerbside to setback concentrations was 2.1 for NO_x_, 1.6 for NO_2_ and 1.1 for PM_10_ (relative to Station 1) based on the fixed sites. A larger number of studies report the degree to which roadside concentrations are elevated relative to an assumed background level. Making comparisons is somewhat limited, however, by differences in the way the background level is estimated, and is also sensitive to the method and correct specification of the distance of the roadside site to the road. Also, it must be borne in mind that international and even inter-road comparisons may be telling us as much about variability in the background as variability in the subject road’s contribution. Karner et al. (2010) attempted to summarise the kerbside/background ratio for a large number of disparate studies finding values of 1.8 for NO_x_, 2.9 for NO_2_ and 1.3 for PM_10_. In the I-15 Las Vegas study, the ratio for NO_x_ was 1.5. In our study, the predominantly upwind and downwind setback sites reported the same mean concentration so that our ratio is the same using both sites. Background NO_x_ concentrations appeared to be higher in our study (~90 μg m^−3^) compared to Las Vegas (~60 μg m^−3^). This may be related to a higher urban background emission density in the south Auckland area. The higher kerbside to setback ratio in our study is consistent with higher NO_x_ emission factors in Auckland, and/or our roadside site being closer to the emission source, as described above.

### Source contribution to peak PM_10_ concentrations

During the campaign, 24-h average PM_10_ exceeded 25 μg m^−3^ at all three of our operating monitoring sites on five occasions. The same dates corresponded to four out of the five highest 24 h averages recorded during the campaign at five permanent Auckland Council monitoring sites ranging from 5 to 32 km from the study area. These observations indicate that the peak roadside concentrations of particulate matter were predominantly related to regional scale reduced dispersion conditions. During the five 24-h periods during which peak concentrations were measured, the difference in PM_10_ between the three study sites remained small throughout the day, and hourly concentrations peaked a few hours either side of midnight, when traffic on the motorway was rapidly falling towards a minimum. This suggests that during times of peak concentrations, the contribution of the motorway to PM_10_ concentrations is small compared to a different source much larger both spatially and in terms of emission rates. That source is most likely to be domestic wood-burning for home heating.

### Commentary on particulate matter

We estimated that emissions from the Auckland Southern Motorway contributed a 1.8-μg m^−3^, or 10 % increase, on average, in PM_10_ concentrations at the roadside, relative to the western setback site. We also estimated that on days when peak 24 h PM_10_ concentrations were observed, the absolute increment was little different at 2.1 μg m^−3^, but that the relative contribution was reduced to 7 %. These values are below those summarised by Karner et al. (2010). However, fair comparison is very difficult to achieve as many of the studies referenced by Karner et al. (2010) featured much shorter campaigns than our study, a wide range of measurement technologies and a wide range of traffic conditions. The contributions estimated are of a similar order of magnitude as the precision of the instruments we deployed (the beta attenuation monitor), i.e. 2 μg m^−3^ over 24 h. Uncertainties in our estimate also arise from the difficulty in assuring that two of our key study design assumptions—that there are no significant sources or sinks between our three fixed monitoring sites and that concentrations measured at our upwind site are representative of air masses arriving at our downwind sites—are valid for PM_10_. The fact that our three sites cannot be on the same trajectory for all wind directions, and that we are monitoring in an urban area with local roads, homes and businesses, introduces the possibility that our upwind site is over-estimating ‘background’ concentrations and that the increment at the downwind site attributed to the motorway only may be due to other sources. We are unable at present to determine whether these errors introduce a net positive or negative error to the estimation of motorway contribution.

### Further results

Further detailed results from this study are presented elsewhere. Estimates of ultrafine particle number concentrations, based on the NO_x_ concentrations reported in this paper, and a consideration of environmental justice, are presented in Pattinson et al. (2014b). Analysis and visualisation of data captured concurrently across the study area using a mobile platform are presented in Pattinson et al. (2014a). Data from the three continuous sites are used to train and validate a semi-empirical model to apportion roadside air quality to highway and background sources in Elangasinghe et al. (2014). A further paper considers the use of data captured in this study as input to a microenvironmental personal exposure model to explore variability in the contribution of the highway to total exposure across the study area (Pattinson et al. *in review*).

### Conclusions

The data presented here from 8 weeks of continuous measurement at three sites at or near a major highway has allowed the calculation of the contribution of that highway to local air quality.

The average roadside increment was calculated to be 1.8, 7.2 and 101 μg m^−3^ for PM_10_, NO_2_ and NO_x_, respectively, relative to a predominantly upwind setback site, and −0.1, 9.4 and 99 μg m^−3^ for PM_10_, NO_2_ and NO_x_, respectively, relative to a downwind setback site. The contribution of the motorway to PM_10_ was difficult to distinguish due to interference from domestic heating sources and because the increments calculated were of a similar order to the precision of the beta attenuation monitors used.

These results may be compared with other studies with caution, bearing in mind differences in the precise distance of the monitor from the road lanes, measurement technologies, meteorology and climate, vehicle fleet makeup and differences in the way background concentrations are estimated.

Future analysis of this dataset could include more detailed data-mining, exploring the larger dataset (i.e. not limited to the period when all three sites were operating), a more detailed comparison with the I-15 study and the contribution of minor roads to local air quality.
